# The Effects of Foliar Supplementation of Silicon on Physiological and Biochemical Responses of Winter Wheat to Drought Stress during Different Growth Stages

**DOI:** 10.3390/plants12122386

**Published:** 2023-06-20

**Authors:** Dongfeng Ning, Yingying Zhang, Xiaojing Li, Anzhen Qin, Chao Huang, Yuanyuan Fu, Yang Gao, Aiwang Duan

**Affiliations:** Key Laboratory of Crop Water Use and Regulation, Ministry of Agriculture and Rural Affairs, Institute of Farmland Irrigation Research, Chinese Academy of Agricultural Sciences, Xinxiang 453002, China; ningdongfeng@caas.cn (D.N.); zhangyingying@caas.cn (Y.Z.); 19913857112@163.com (X.L.); qinanzhen@caas.cn (A.Q.); 18703737321@163.com (C.H.); fyycaas@gmail.com (Y.F.)

**Keywords:** silicon, wheat, drought, photosynthesis, antioxidant defense

## Abstract

Drought is one of the major environmental stresses, resulting in serious yield reductions in wheat production. Silicon (Si) has been considered beneficial to enhancing wheat resistance to drought stress. However, few studies have explored the mediated effects of foliar supplementation of Si on drought stress imposed at different wheat growth stages. Therefore, a field experiment was carried out to investigate the effects of Si supplementation on the physiological and biochemical responses of wheat to drought stress imposed at the jointing (D-jointing), anthesis (D-anthesis) and filling (D-filling) stages. Our results showed that a moderate water deficit markedly decreased the dry matter accumulation, leaf relative water content (LRWC), photosynthetic rate (Pn), stomatal conductance (Sc), transpiration rate (Tr) and antioxidant activity [peroxidase (POD), superoxide dismutase (SOD) and catalase (CAT)]. On the contrary, it remarkably increased the content of osmolytes (proline, soluble sugar, soluble protein) and lipid peroxidation. The grain yields of D-jointing, D-anthesis and D-filling treatments were 9.59%, 13.9% and 18.9% lower, respectively, compared to the control treatment (CK). However, foliar supplementation of Si at the anthesis and filling stages significantly improved plant growth under drought stress due to the increased Si content. Consequently, the improvement in antioxidant activity and soluble sugar, and the reduction in the content of ROS, increased the LRWC, chlorophyll content, Pn, Sc and Tr, and ultimately boosted wheat yield by 5.71% and 8.9%, respectively, in comparison with the non-Si-treated plants subjected to water stress at the anthesis and filling stages. However, the mitigating effect of Si application was not significant at the jointing stage. It was concluded that foliar supplementation of Si, especially at the reproductive stage, was effective in alleviating drought-induced yield reduction.

## 1. Introduction

Drought is one of the most significant abiotic stresses in agriculture, posing a severe threat to plant growth, development and yield around the world [1]. Drought disrupts the integrity of the membrane, the balance between the production of reactive oxygen species (ROS) and the antioxidant system, and water–nutrient relationships, thus impacting photosynthetic activity in a number of crops [2]. Wheat (*Triticum aestivum* L.) is one of the three major cereals widely grown on the planet. The deficit in irrigated water is a serious problem impacting wheat production in arid and semi-arid areas [3,4,5]. Drought stress causes annual global wheat yield losses of 20% [6]. Thus, effective methods for increasing wheat resistance to drought stress are urgently required to ensure food security. 

Silicon (Si) has been considered as a beneficial element for plant growth and development, especially under biotic or abiotic stresses [7,8]. Numerous studies have reported that Si was useful in strengthening drought tolerance in some Si-accumulated plants such as rice [9], wheat [10], maize [11] and sorghum [12], as well as in other plants such as oilseed rape [13], lentil [14], mango [15] and tomato [16]. The mechanisms by which Si regulates plant resistance to drought stress are mainly associated with crop root growth and water uptake [17,18]. It has been reported that Si supplementation improves osmolyte content [19], enhances the activities of antioxidant enzymes [20] and modifies photosynthetic activity and gas exchange [20]. Although Si is abundant in soil, most types of Si are not in a plant-available form [21]. Plants absorb Si only as silicic acid [22]. Many studies have demonstrated that the exogenous foliar supplementation of Si is effective in mitigating drought damage in various plant species, such as wheat [23], pearl millet [24] and chestnut [25]. Drought stress impacts wheat growth at any growth stage, but crop responses to Si supplementation vary at different growth stages [4]. As a result, the regulated effect of Si on drought stress varies at different growth stages of crops [11,19]. During the whole growth period of wheat, the jointing, anthesis and filling stages are key to the crop water requirement. Thus, we conducted an experiment across the entire wheat life cycle in order to (1) explore the physiological and biochemical responses of winter wheat to drought stress at the jointing, anthesis and filling stages, and (2) to identify the effects of foliar supplementation of Si on improving plant growth, physiological and biochemical characteristics, and grain yield in wheat under drought stress at the jointing, anthesis and filling stages. The results are expected to be beneficial to the sustainable production of winter wheat in arid and semi-arid areas.

## 2. Results

### 2.1. Plant Growth

**Figure 1:**
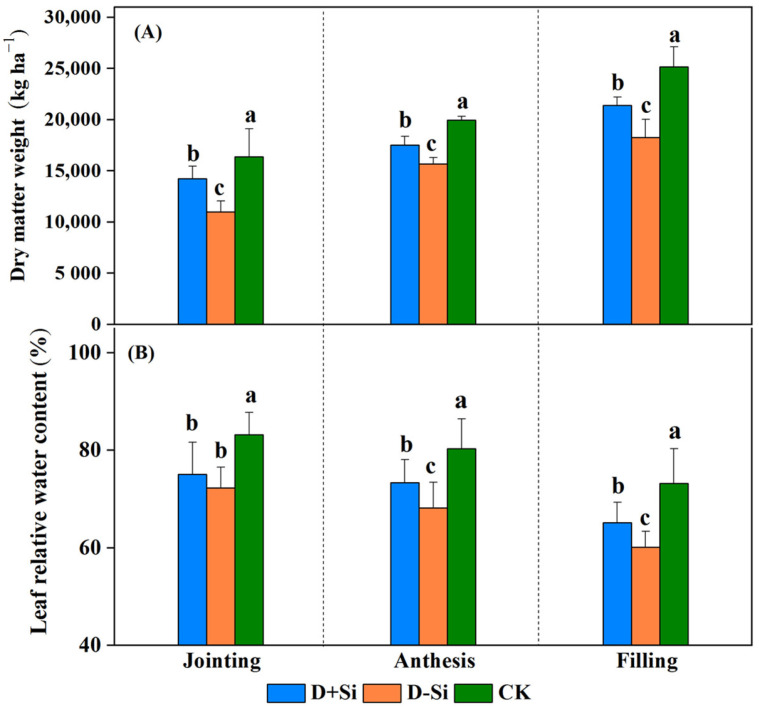
Effect of foliar application of Si fertilizer on leaf relative water content (**A**) and dry matter (**B**) of wheat at jointing, anthesis and filling stages under drought and normal conditions. +Si, Si addition; -Si, no Si addition; D, drought stress; CK, normal irrigation throughout all stages. Data are the means ± standard deviation (SD) of three replicates. Different letters (a, b, c) above the bars indicate statistical significance (*p* ≤ 0.05).

Eight days of moderate water deficit imposed at the jointing, anthesis and filling stages markedly inhibited dry matter accumulation and the leaf relative water content of the wheat compared with normal irrigation (CK) (*p* ≤ 0.05) (Figure 1). Foliar supplementation of Si fertilizer significantly improved the dry matter weight and leaf relative water content (LRWC) compared with non-Si applied treatments under drought stress imposed at the anthesis and filling stages (*p* ≤ 0.05). Under drought stress, the dry matter accumulation of non-Si applied treatments decreased by 21.5% and 27.4%, respectively, at the anthesis and filling stages, compared with the normal irrigation treatments, while the decrease levels were decreased by 13.0%, 12.2% and 15.0%, respectively, in the Si applied treatments. Meanwhile, the LRWC of the D+Si treatments were 7.62% and 8.49% higher than those of the D-Si treatments at the anthesis and filling stages, respectively. However, the increases in dry matter accumulation and LRWC with Si applied were not significant under drought stress at the jointing stage.

### 2.2. Photosynthesis and Chlorophyll Content

**Figure 2:**
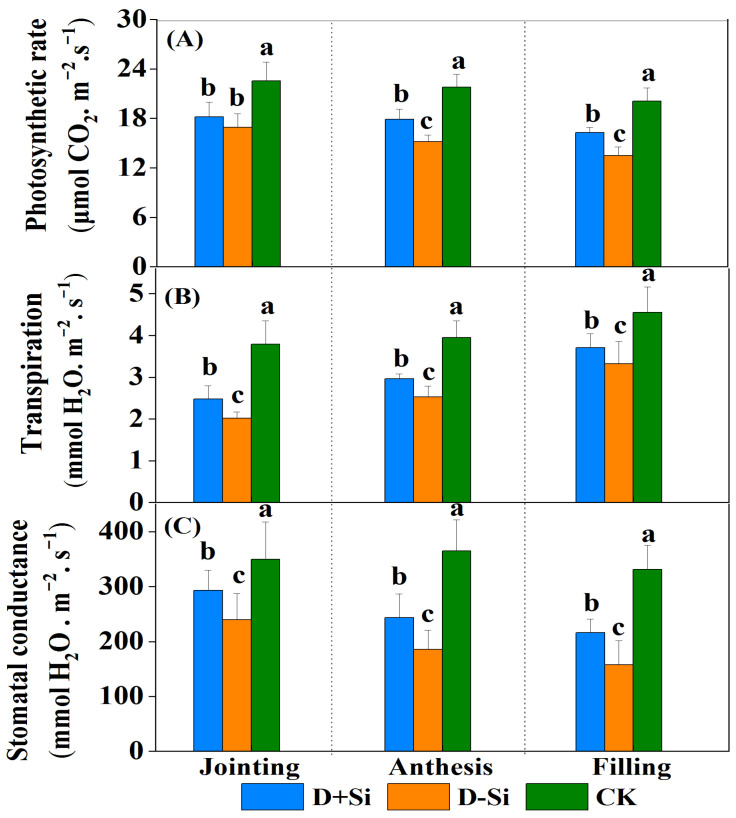
Effect of foliar application of Si fertilizer on photosynthetic rate (**A**), stomatal conductance (**B**) and transpiration (**C**) of wheat at jointing, anthesis and filling stages under drought and normal conditions. +Si, Si addition; -Si, no Si addition; D, moderate drought stress; CK, normal irrigation throughout all stages. Data are the means ± standard deviation (SD) of three replicates. Different letters (a, b, c) above the bars indicate statistical significance (*p* ≤ 0.05).

Water stress imposed at the jointing, anthesis and filling stages remarkably reduced the plant photosynthetic rate (Pn), stomatal conductance (Sc) and transpiration rate (Tr) in comparison with normal irrigation (*p* ≤ 0.05) (Figure 2),whereas foliar supplementation with Si significantly increased the Pn, Tr and Sc of the wheat leaves compared with non-Si-treated plants under water deficit conditions across the three stages (except the photosynthetic rate at the jointing stage).

**Figure 3:**
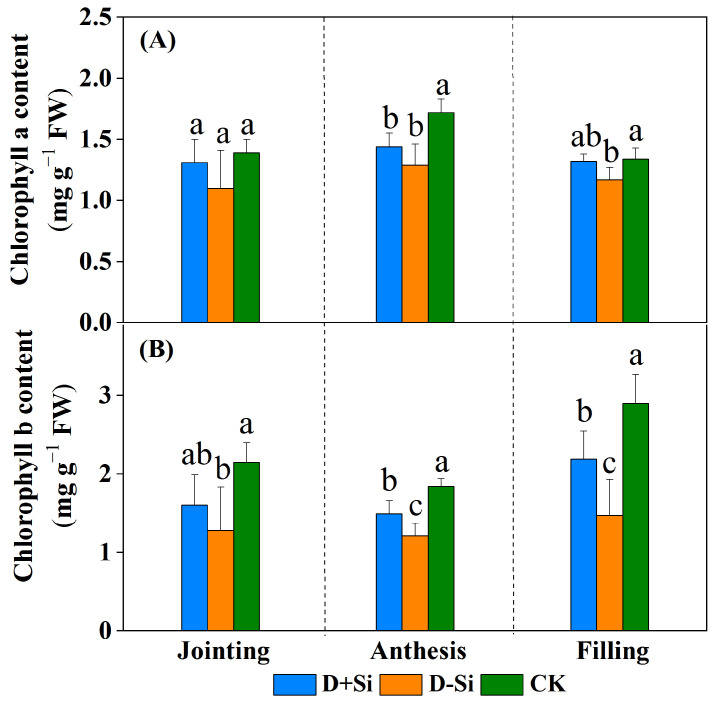
Effect of foliar application of Si fertilizer on chlorophyll a (**A**) and chlorophyll b (**B**) content in wheat leaves at jointing, anthesis and filling stages under drought and normal conditions. +Si, Si addition; -Si, no Si addition; D, drought stress; CK, well-watered control conditions throughout all stages. Data are the means ± standard deviation (SD) of three replicates. Different letters (a, b, c) above the bars indicate statistical significance (*p* ≤ 0.05).

The chlorophyll a and chlorophyll b content was also significantly reduced under water deficit imposed at the jointing, anthesis and filling stages compared with CK treatments (*p* ≤ 0.05), with the exception of the jointing stage (*p* ≤ 0.05) (Figure 3). Foliar supplementation of Si significantly increased the chlorophyll b content compared with non-Si applied treatments under drought stress (*p* ≤ 0.05), but the increase in chlorophyll a content was not significant between the treatments of Si and non-Si application across the three stages (*p* > 0.05).

### 2.3. Osmotic Solute Content

**Figure 4:**
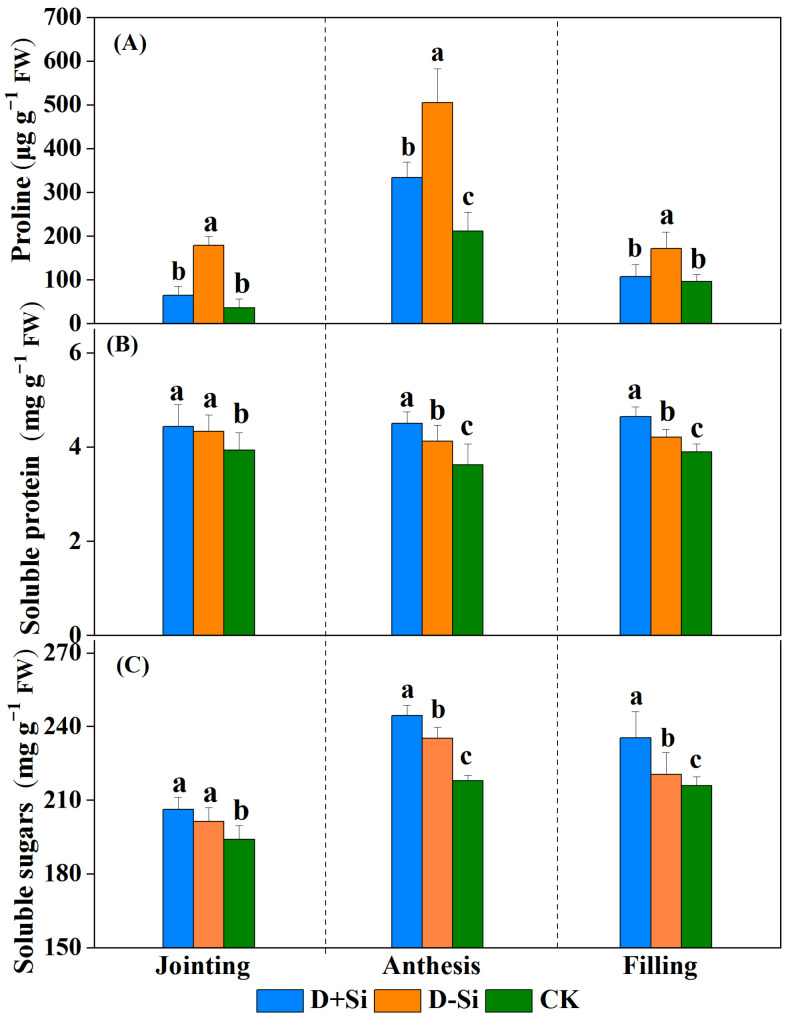
Effect of foliar application of Si fertilizer on proline (**A**), soluble protein (**B**) and soluble sugar content (**C**) in wheat leaves at jointing, anthesis and filling stages under drought and normal conditions. +Si, Si addition; -Si, no Si addition; D, drought stress; CK, well-watered controls condition. Data are the means ± standard deviation (SD) of three replicates. Different letters (a, b, c) above the bars indicate statistical significance (*p* ≤ 0.05).

The osmotic solute content is shown in Figure 4. The proline, soluble protein and sugar content was significantly increased as a response to drought stress (*p* ≤ 0.05). Si application significantly decreased proline content by 64.0%, 33.9% and 37.5%, respectively, at the jointing, anthesis and filling stages compared with non-Si applied treatment under drought stress (*p* ≤ 0.05) (Figure 4A), whereas the soluble protein and sugar content in the Si treatments was significantly enhanced by 3.92%, 6.78%, and 9.22%, 10.2%, respectively, in comparison with non-Si applied treatment under drought stress imposed at the anthesis and filling stages (*p* ≤ 0.05) (Figure 4B,C). The increase in the soluble protein and sugar content between the Si and non-Si treatments was not significant under drought stress at the jointing stage. 

### 2.4. Superoxide Radicals and MDA Content

**Figure 5:**
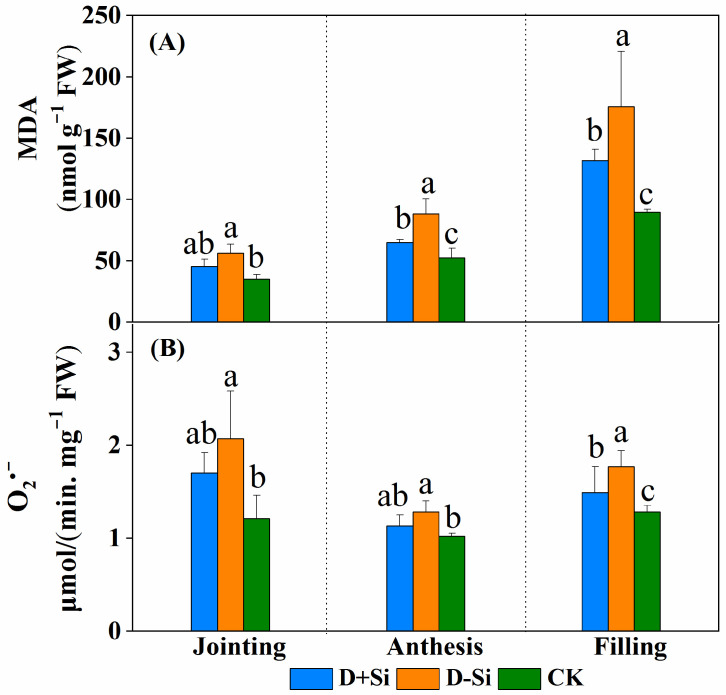
Effect of foliar application of Si fertilizer on malondialdehyde (MDA) (**A**) and superoxide radical (O_2_^·−^) content (**B**) in wheat leaves at jointing, anthesis and filling stages under drought and normal conditions. +Si, Si addition; -Si, no Si addition; D, drought stress; CK, well-watered controls condition. Data are the means ± standard deviation (SD) of three replicates. Different letters (a, b, c) above the bars indicate statistical significance (*p* ≤ 0.05).

Under water deficit conditions, malondialdehyde (MDA) content and superoxide radical (O_2_^·−^) in wheat leaves significantly increased in comparison with the normal irrigation treatment across the three stages (*p* ≤ 0.05) (Figure 5). Under drought conditions, foliar supplementation of Si fertilizer markedly decreased the MDA and O_2_^·−^ content in the leaves. In detail, the MDA content was decreased by 19.1%, 26.2% and 25.0%, respectively and the O_2_^·−^ content was decreased by 17.9%, 11.7% and 15.8%, respectively, at the jointing, anthesis and filling stages compared with the corresponding non-Si applied treatments.

### 2.5. Antioxidant Enzyme Activity

**Figure 6:**
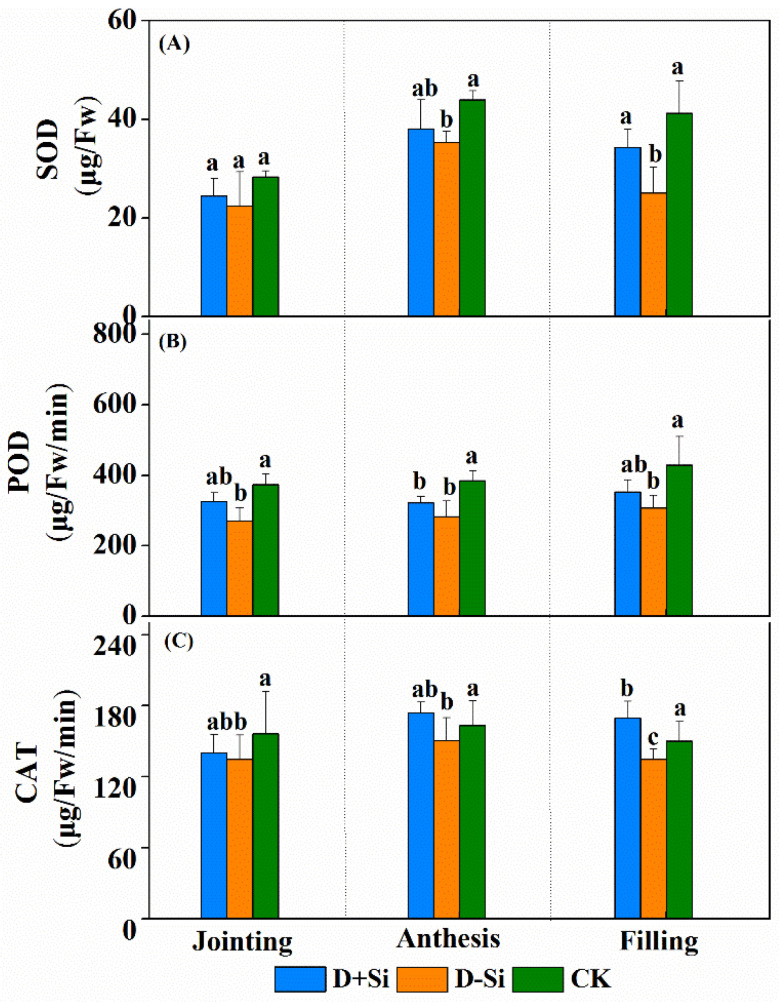
Effect of foliar application of Si fertilizer on superoxide dismutase (SOD) (**A**), peroxidase (POD) (**B**) and catalase (CAT) (**C**) activities content in wheat leaves at jointing, anthesis and filling stages under drought and normal conditions. +Si, Si addition; -Si, no Si addition; D, drought stress; CK, well-watered controls condition. Data are the means ± standard deviation (SD) of three replicates. Different letters (a, b, c) above the bars indicate statistical significance (*p* ≤ 0.05).

Water deficit imposed at the jointing, anthesis and filling stages significantly reduced the activities of superoxide dismutase (SOD), peroxidase (POD) and catalase (CAT) (except SOD at jointing stage) (*p* ≤ 0.05) (Figure 6). The SOD, POD and CAT activities were significantly enhanced by foliar supplementation of Si fertilizer under water stress at the filling stage, while the increases of SOD, POD and CAT activities at the jointing and anthesis stages were not significant.

### 2.6. Grain Yield

Moderate water deficit generally caused a remarkable decrease in wheat yields, with the grain yields of the D-jointing-Si, D-anthesis-Si and D-filling-Si treatments 9.59%, 13.9% and 18.9% lower than the CK treatments, respectively (Table 1). With the foliar supplementation of Si fertilizer, the grain yields of the D-jointing, D-anthesis and D-filling treatments were 4.37%, 5.71% and 8.9% higher, respectively, than the corresponding non-Si treatments. Water deficit imposed at the jointing and anthesis stages significantly decreased the straw biomass compared with normal irrigation (*p* ≤ 0.05); the decrease in D-filling treatment was not significant. Contrary to the pattern in wheat yield, foliar supplementation of Si fertilizer showed little impact on the straw biomass of the wheat under drought conditions, regardless of the growth stage. The difference in the wheat harvest index was not significant across the D-jointing, D-anthesis and CK treatments, regardless of whether or not Si application had occurred. However, drought stress imposed at the filling stage significantly decreased the harvest index, while foliar supplementation of Si fertilizer remarkably reduced the decline (Table 1).

### 2.7. Si concentration in Plant Tissues and Soil

**Figure 7:**
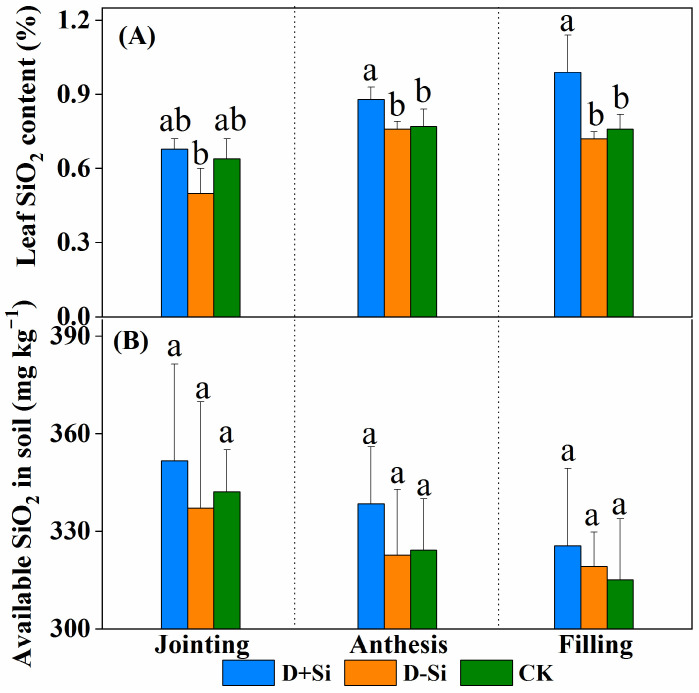
Effect of foliar application of Si fertilizer on Si concentration in wheat leaves (**A**) and soil (**B**) at jointing, anthesis and filling stages under drought and normal conditions. +Si, Si addition; -Si, no Si addition; D, drought stress; CK, well-watered controls condition. Data are the means ± standard deviation (SD) of three replicates. Different letters (a, b, c) above the bars indicate statistical significance (*p* ≤ 0.05).

Foliar supplementation of Si fertilizer remarkably increased the Si content in the wheat leaves. The highest Si content in the leaves was observed at the filling stage (Figure 7). However, Si application had no significant impact on the plant-available Si concentration in the soil. The plant-available Si concentration in the soil decreased gradually with the growth of the wheat plant.

### 2.8. Correlation Analysis

**Figure 8:**
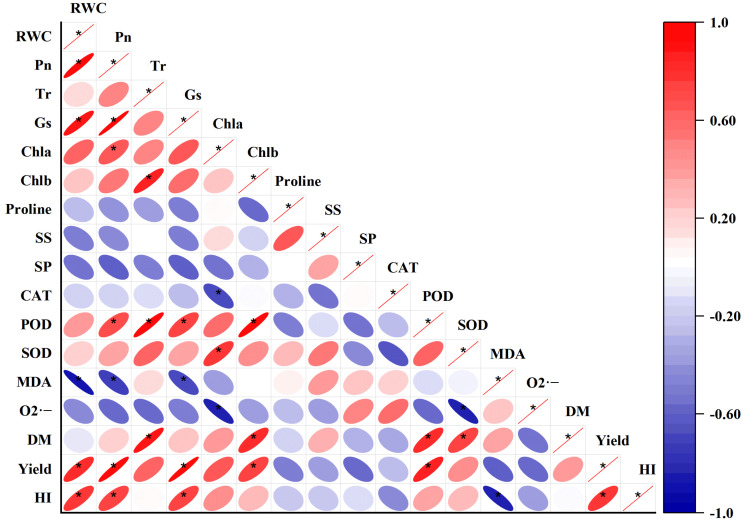
Pearson correlation analysis among yield and selected growth and physiological parameters measured at different growth stages. Red and blue represent positive and negative correlations, respectively. The deeper the color, the smaller the shape and the stronger the correlations. RWC, relative water content; Pn, photosynthetic rate; Tr, transpiration; Gs, stomatal conductance; Chla, chlorophyll a; Chlb, chlorophyll b; SS, soluble sugar; SP, soluble protein; DM, dry matter; HI, harvest index. * Significant at *p* ≤ 0.05.

The results of correlation analysis revealed that RWC, Pn, Chlb and POD were considered to be the major wheat yield indexes attributed to their observably positive relations with yield (Figure 8). Significant relationships were found among RWC, Pn, Gs and Chla. However, proline was negatively correlated with RWC, Pn, Tr, Gs and yield, although the correlation coefficients were not significant.

## 3. Discussion

### 3.1. Effect of Si on Plant Growth, and Photosynthesis under Water Stress

Drought is one of the major abiotic stress factors, which can result in insufficient water uptake for plant growth and thus negatively influence plant morphological, physiological and biochemical characteristics [8]. Dry matter accumulations are an important morphological indicator of plant status under stress condition. In this work, the dry matter accumulations of the drought-stressed plants were significantly decreased compared with the normal plants, while the decrease levels were significantly reduced by foliar supplementation of Si fertilizer under water stress at the anthesis and filling stages (*p* ≤ 0.05) (Figure 1). This finding is in line with the research of Meunier et al. (2017) in wheat [10], Xu et al. (2022) in maize [19] and Wasaya et al. (2022) in pearl millet [24]. The decline in biomass accumulation ultimately affects the yield (Table 1). Maintaining a high photosynthetic rate is crucial to plant growth and yield production under water stress. Regardless of the growth period, moderate drought stress significantly restricted the wheat photosynthetic rate in this study (Figure 2), whereas Si supplementation remarkably alleviated the decline of the photosynthetic rate at the anthesis and filling stages under drought stress and thus improved plant growth. The enhancement of the photosynthetic rate mediated by Si supplementation could be ascribed to several factors.

Firstly, silicon concentrations in leaves were significantly increased by Si supplementation at the anthesis and filling stages (Figure 7A). The higher Si concentrations caused a high accumulation of silicophytolith in the leaves under drought stress, which provided better support to the leaves and was beneficial to photosynthesis [10,12]. Secondly, the transpiration rate (Tr) and stomatal conductance (Sc) are two major factors affecting plant–water relationships [26]. There are two viewpoints on the improvement of leaf water status mediated by Si supplementation. Earlier studies indicated that Si deposition beneath the cuticle may decrease cuticular transpiration and thus reduce water loss [27]. However, recently, most researchers observed that Si addition increased the plant transpirational rate and led to better stomatal structures under water deficit due to enhanced root water uptake and/or transport [16,28,29]. In the present work, the results were consistent with the point that Si may be mediated by root water absorption and transport, maintaining water balance in response to water stress. Liu et al. (2014) demonstrated that silicon-induced augmentation of Tr and Sc was related to the increase in root hydraulic conductance resulting from the increase in aquaporins under water stress [18]. Mastalerczuk et al. (2023) reported that Si application enhanced the allocation of carbon to the roots to develop the fine network under drought stress [30].

Thirdly, the maintenance of the chlorophyll content of the Si-treated plant leaves promoted photosynthesis (Figure 3). In this study, drought significantly decreased the chlorophyll a and chlorophyll b content, which was attributed to excessive accumulation of ROS and higher oxidative damage (Figure 5). The increase in chlorophyll b content induced by Si application may be due to Si-mediated increases in the antioxidant activity (Figure 7) [31]. Fourthly, the activities of the photosynthetic enzymes, such as RuBisCo and PEPCase activities were increased by Si addition under water deficit conditions [19,32]. Fifthly, maintaining chloroplast and thylakoid integrity resulted from the lower levels of oxidative stress [33]. Additionally, Si application may be effective in the absorption, transformation and transfer of light energy by changing the thylakoid membrane protein components under water deficit conditions [9].

However, in the present study, the increases of plant dry matter accumulations, LRWC and photosynthetic rate induced by Si application were not significant compared with non-Si-treated plants under water deficit imposed at the jointing stage (Figure 1 and Figure 2A). Gong et al. (2008) reported that under drought stress, supplementation of Si increased plant water potential at the filling stage, but which showed little impact at booting stage [34]. Ma et al. (2016) also indicated that application of silicon alleviated photosynthesis damage caused by water deficit in the later growth stages of wheat [31]. This might be attributed to that plant-available silicon in soil was sufficient for wheat growth at the jointing stage (Figure 7B), so the effect of Si supplementation on Si accumulations in the leaves was not significant (Figure 7A). The results also recommended that Si application was more effective on enhancing wheat resistance to drought at the reproductive stage.

### 3.2. Effect of Si on Osmotic Adjustment and Antioxidant Defense under Water Stress

Plants accumulate solutes (i.e., proline, glucose, glycine-betaine and soluble sugar) to maintain optimum water content under drought [35]. In this work, proline, soluble protein and soluble sugar content were significantly enhanced under water stress (*p* ≤ 0.05) (Figure 4). Proline acts as a signaling molecule under stressful environment [36]. Foliar supplementation of Si significantly decreased proline content under water stress, (Figure 4A), and negative correlations were observed between proline content and yield, RWC, Pn, Tr and Gs (Figure 8). The decline in proline content induced by Si addition indicated the recovery of relative water content during water stress [37]. Pei et al. (2010) also reported that proline content was significantly negatively related with dry weight and leaf chlorophyll content in wheat seedlings [38]. The finding suggested that proline did not play role in osmotic adjustment mediated by Si application under drought stress. However, contrary to the above findings, some other studies reported that proline content was significantly increased by Si application under water stress, indicating that proline is beneficial in osmotic adjustment [19,39]. Soluble sugars are considered as a marker of capacity of osmotic adjustment in response to water stress [40]. In this work, silicon supplementation further increased the soluble sugar and protein content compared with non-Si-treated plants under drought stress (Figure 4B,C). These results were consistent with the findings of Yin et al. (2014) in sorghum [28] and Ning et al. (2020) in maize [11]. The enhancement of the soluble sugar content induced by Si application was primarily attributed to the acceleration of starch hydrolysis and the decline of sugar utilization [19]. On the other hand, some studies found that Si application resulted in a lower soluble sugar concentration, suggesting that Si application decreased the anabolism of soluble sugar under water stress [14]. In conclusion, the mechanisms on the osmotic adjustment mediated by Si addition under water stress are still controversial and need to be further studied at the molecular level.

Water stress breaks the equilibrium between the production of reactive oxygen species (ROS) and the antioxidant system; excessive accumulation of ROS results in protein and lipid peroxidation, reduced membrane stability and generates a high accumulation of MDA [8,41]. Under water stress, plants generate high levels of antioxidants, such as SOD, POD, CAT and APX, to cope with ROS-induced oxidative damage [31]. In this work, drought stress significantly increased the MDA and O_2_^·−^ content in wheat leaves, but downregulated the activities of SOD, POD and CAT, while Si supplementation markedly enhanced the SOD, POD and CAT activities compared to non Si-treated leaves, thus decreasing the MDA and O_2_^·−^ concentrations in the plants. The results were in line with study of Shi et al. (2014) in tomato [42], Biju et al. (2017) in lentil [14] and Parveen et al. (2019) in maize [37]. Silicon application increased the transcription of TaSOD, TaCAT and TaAPX in the plants under drought stress, which indicated that Si played a crucial role in the coordinated transcriptional regulation of multiple antioxidant defenses in response to water stress [31].

### 3.3. Effect of Si on Wheat Biomass and Yield under Water Stress

Drought stress occurring at any growth stage limits wheat growth, while the anthesis and filling stages were more sensitive to drought stress, resulting in substantial yield reduction [4,43,44]. Our study confirmed that drought stress imposed at the filling stage caused the largest yield decline, followed by the anthesis stage and then the jointing stage (Table 1). The harvest index (HI) followed the same order. The HI of the D-filling treatment was significantly lower than the others. The supplementation of Si efficiently decreased the wheat yield reduction induced by water stress, especially at the filling stage. Correlation analysis showed that RWC, Pn, Chlb and POD were significantly positively related with yield (Figure 8). The results were similar to the study of Bukhari et al. (2021) [45], who also reported that the application of Si markedly enhanced wheat growth and grain yield at the anthesis and filling stages under water stress. Lavinsky et al. (2016) reported that Si application during the rice reproductive stage was more effective on yield production [46]. Xu et al. (2022) also suggested that the effect of Si application on alleviating damage induced by drought stress was better at the maize tasseling stage than that at the jointing stage [19]. Thus, it was concluded that the positive role of Si in mitigating drought-induced yield decline was more efficient when the water deficit occurred during the reproductive stage.

## 4. Materials and Methods

### 4.1. Experimental Site and Treatments

A field experiment was carried out in a lysimeter facility equipped with a rainproof shelter from October 2019 to May 2020 in winter wheat season at the Chinese Academy of Agricultural Sciences Experimental Station (35°18′ N, 113°54′ E, 80 m altitude), located in Qiliying town, Xinxiang city, Henan Province, P.R. China. This area has a semi-humid monsoon climate. The lysimeter facility comprised 24 lysimeters (3 m × 2.2 m × 1.5 m, length × width × depth), filled with sandy loam soil. The detailed descriptions of the lysimeters were presented in Ning et al. (2019) [47]. The texture of the soil filling the lysimeters had an average bulk density of 1.42 g cm^−3^ and a field capacity of 24.5% in the 0–100 cm soil profile. The soil’s basic chemical properties in the top 20 cm were soil organic matter 12.9 g kg^−1^, total nitrogen (N) 0.76 g kg^−1^, inorganic N 26.5 mg kg^−1^, Olsen-P (P) 12.6 mg kg^−1^, available (K) 212.3 mg kg^−1^, available (Si) mg kg^−1^ and pH 8.23. Summer maize was the previous crop in the lysimeters. 

The experiment set two Si fertilization rates and three stages of drought stress. The two Si fertilization levels included 0 Si (D-Si) and 2 mM Si (D+Si). Si fertilizer was applied as Na_2_SiO_3_·9H_2_O. Three growth stages, namely, the jointing, anthesis and filling stages, were independently placed under moderate drought stress for 8 d, which corresponded to 50% of field capacity. Sufficient water supply (80% field capacity) during the whole growth stages was set as the control treatment (CK). Seven treatments were arranged in a randomized block design with three replications. Water stress was induced by withholding irrigation. Insentek sensors (Beijing Oriental Ecological Technology Ltd., Co., Beijing, China) were installed in each lysimeter to monitor soil volumetric water content (cm^3^· cm^−3^) to a depth of 100 cm [48]. Silicon solution was sprayed once every two days during the drought stress days at the jointing, anthesis and filling stages, and the D-Si and CK treatments were sprayed with an equal amount of distilled water. Irrigation was applied normally during the rest of the growth time.

The variety of winter wheat “Zhoumai 22” (a dominant cultivar in the North China Plain) was planted (row spacing 20 cm, seeding rate 225 kg hm^−2^) on 16 October 2020 and harvested on 20 May 2021. A basal fertilization was applied before seeding, including 108 kg ha^−1^ N (Urea, 46% N), 100 kg ha^−1^ P_2_O_5_ (superphosphate, 12% P_2_O_5_) and 100 kg K_2_O ha^−1^ (potassium sulfate, 50% K_2_O) for each treatment. Nitrogen fertilizer at the rate of 72 kg ha^−1^ N (Urea, 46% N) was applied at the wheat jointing stage. The irrigation method was drip irrigation, composed of four drip tapes, a water meter, a fertigation tank and a sand filter. Nitrogen fertilizer was applied by drip fertigation system.

### 4.2. Sampling and Measurements

- Leaf samples

Leaf samples (the second upper fully expanded leaves at the jointing stage and the flag leaves at the anthesis and filling stages) were collected from each plot on the last day of each drought stress stage. These leaf samples were shortly afterwards frozen in liquid nitrogen and then stored at −80 °C prior to physiological analysis. The selected analytical protocols were described in Ning et al. (2020) [11].

- Dry weight of above biomass and grain yield

On the last day of drought stress at the jointing, anthesis and filling stages and at grain maturity, 0.5 m wheat plants were cut to the ground. The fresh samples were primarily dried for 0.5 h in an oven at 105 °C and then dried at 75 °C to a constant weight. Subsequently, the leaf samples were ground and passed through a 0.5-mm sieve for Si analysis. 

At the maturity stage, 2 m^2^ of undisturbed wheat was collected for yield measurement. The grains were weighed after natural air drying.

- Photosynthetic rate, transpiration rate and stomatal conductance

On the last day of each drought stress period, the second upper leaves (fully expanded) at the jointing stage and the flag leaves at the anthesis and filling stages were used to measure photosynthetic rate, transpiration rate and stomatal conductance. Leaves were measured between 9:00 and 11:00 a.m. using a portable photosynthesis system (LI-6400, LI-COR., Lincoln, NE, USA).

### 4.3. Statistical Analysis

One-way analysis of variance (ANOVA) was performed to detect the significant differences between the means of the different treatments, tested by Tukey’s least significant difference (LSD) at a significance level of *p* ≤ 0.05. Pearson correlation analyses were performed to investigate the relationships among yield and physiological indices. SPSS 18.0 (SPSS Inc., Chicago, IL, USA) was used to conduct statistical analyses, and graphs were drawn using Origin 2022 software (OriginLab, Northampton, MA, USA). 

## 5. Conclusions

The present study demonstrated that moderate drought stress imposed at the jointing stage markedly decreased RWC, chlorophyll content, photosynthesis and antioxidant activity, but increased lipid peroxidation, thus resulting in a significant decline in wheat yield. Water deficit during the filling stage resulted in the largest yield reduction, followed by the anthesis stage. Foliar supplementation of Si at the anthesis and filling stages significantly enhanced plant growth under drought stress through increased Si content, improved antioxidant activity and soluble sugar and reduced ROS, and thus increased RWC and chlorophyll content, promoting photosynthesis and ultimately increasing wheat yield. It was concluded that foliar supplementation of Si, especially at the reproductive stage, minimized the loss of wheat yield induced by drought stress. Further study is needed to explore the molecular mechanisms of Si-regulated drought tolerance in wheat. 

## Figures and Tables

**Table 1:** The effect of silicon fertilization and drought stress on wheat yield, straw biomass and the harvest index.

Factor	Wheat Yield (kg ha^−1^)	Straw Biomass (kg ha^−1^)	Harvest Index (%)
D-jointing+Si	10,804 ± 354 ^ab^	17,180 ± 780 ^b^	38.6 ± 0.58 ^a^
D-anthesis+Si	10,175 ± 313 ^b^	16,965 ± 612 ^b^	37.5 ± 1.15 ^a^
D-filling+Si	10,421 ± 339 ^bc^	17,750 ± 864 ^ab^	37.0 ± 1.53 ^a^
D-jointing-Si	10,352 ± 116 ^bc^	17,198 ± 381 ^b^	37.6 ± 0.58 ^a^
D-anthesis-Si	9858 ± 694 ^bc^	16,755 ± 665 ^b^	37.0 ± 0.63 ^a^
D-filling-Si	9290 ± 430 ^c^	18,113 ± 1362 ^ab^	33.9 ± 1.53 ^b^
CK	11,450 ± 1119 ^a^	18,840 ± 223 ^a^	37.8 ± 2.08 ^a^

D-jointing, D-anthesis and D-filling means drought stress imposed at wheat jointing, anthesis and filling stages; CK, well-watered controls condition. +Si, Si addition; -Si, no Si addition; D, drought stress; Treatment means followed by the different letters are significantly different at *p* ≤ 0.05.

## Data Availability

Not applicable.
